# Safety and Efficacy of Co-Trimoxazole for Treatment and Prevention of *Plasmodium falciparum* Malaria: A Systematic Review

**DOI:** 10.1371/journal.pone.0056916

**Published:** 2013-02-22

**Authors:** Christine Manyando, Eric M. Njunju, Umberto D’Alessandro, Jean-Pierre Van geertruyden

**Affiliations:** 1 Department of Public Health, Tropical Diseases Research Centre, Ndola, Zambia; 2 Department of Public Health, Institute of Tropical Medicine, Antwerp, Belgium; 3 Medical Research Council Unit, Fajara, The Gambia; 4 International Health, University of Antwerp, Belgium; 5 Department of Biomedical Sciences, Tropical Diseases Research Centre, Ndola, Zambia; Kenya Medical Research Institute (KEMRI), Kenya

## Abstract

**Introduction:**

Cotrimoxazole (CTX) has been used for half a century. It is inexpensive hence the reason for its almost universal availability and wide clinical spectrum of use. In the last decade, CTX was used for prophylaxis of opportunistic infections in HIV infected people. It also had an impact on the malaria risk in this specific group.

**Objective:**

We performed a systematic review to explore the efficacy and safety of CTX used for P.falciparum malaria treatment and prophylaxis.

**Result:**

CTX is safe and efficacious against malaria. Up to 75% of the safety concerns relate to skin reactions and this increases in HIV/AIDs patients. In different study areas, in HIV negative individuals, CTX used as malaria treatment cleared 56%–97% of the malaria infections, reduced fever and improved anaemia. CTX prophylaxis reduces the incidence of clinical malaria in HIV-1 infected individuals from 46%–97%. In HIV negative non pregnant participants, CTX prophylaxis had 39.5%–99.5% protective efficacy against clinical malaria. The lowest figures were observed in zones of high sulfadoxine-pyrimethamine resistance. There were no data reported on CTX prophylaxis in HIV negative pregnant women.

**Conclusion:**

CTX is safe and still efficacious for the treatment of P.falciparum malaria in non-pregnant adults and children irrespective of HIV status and antifolate resistance profiles. There is need to explore its effect in pregnant women, irrespective of HIV status. CTX prophylaxis in HIV infected individuals protects against malaria and CTX may have a role for malaria prophylaxis in specific HIV negative target groups.

## Introduction

Worldwide, malaria is one of the most important causes of morbidity and mortality, with children under five years of age and pregnant women being the most severely affected groups. [1] An estimated 3·3 billion people were at risk of malaria in 2010. [2] Of all geographical regions, populations living in sub-Saharan Africa (SSA) have the highest risk of acquiring malaria; in 2010, 81% and 91% of malaria cases and deaths occurred in the World Health Organisation (WHO) African Region [1].

Artemisinin-based combination therapy (ACT) is currently the mainstay of malaria treatment in both children and adults, while in pregnancy it can be used only in the second and third trimester. [3] Pregnant women are more susceptible to malaria infection than other adults, resulting in placental malaria and anaemia and increasing the risk of low birth weight and infant mortality. [4]–[8] Approximately 50 million women living in malaria-endemic areas become pregnant each year, half of them in areas of SSA with stable malaria transmission. The strategies to control malaria during pregnancy rely on case management as well as on a package of preventive measures including insecticide treated nets (ITNs) and intermittent preventive treatment (IPTp) with sulfadoxine-pyrimethamine (SP), a folate inhibitor [9] and as per recommendation of WHO [10]. Malaria prevention is also important for children because of their increased susceptibility to severe illness and death. WHO recommends IPT with SP in infants (IPTi) within the context of the expanded programme of immunisation (EPI) as well as seasonal malaria chemoprevention, previously known as IPT in children (IPTc), with amodiaquine and SP given at regular intervals. This is in addition to the overall recommendations for malaria control that include ITNs, Insecticide Residual Spraying (IRS) and access to prompt diagnosis and treatment for malaria patients. SP is currently the only antimalarial drug used for IPTi or IPTp. However, SP efficacy for treatment of symptomatic malaria has declined over the years, raising concerns about its longevity for IPT [11].

**Figure 1:**
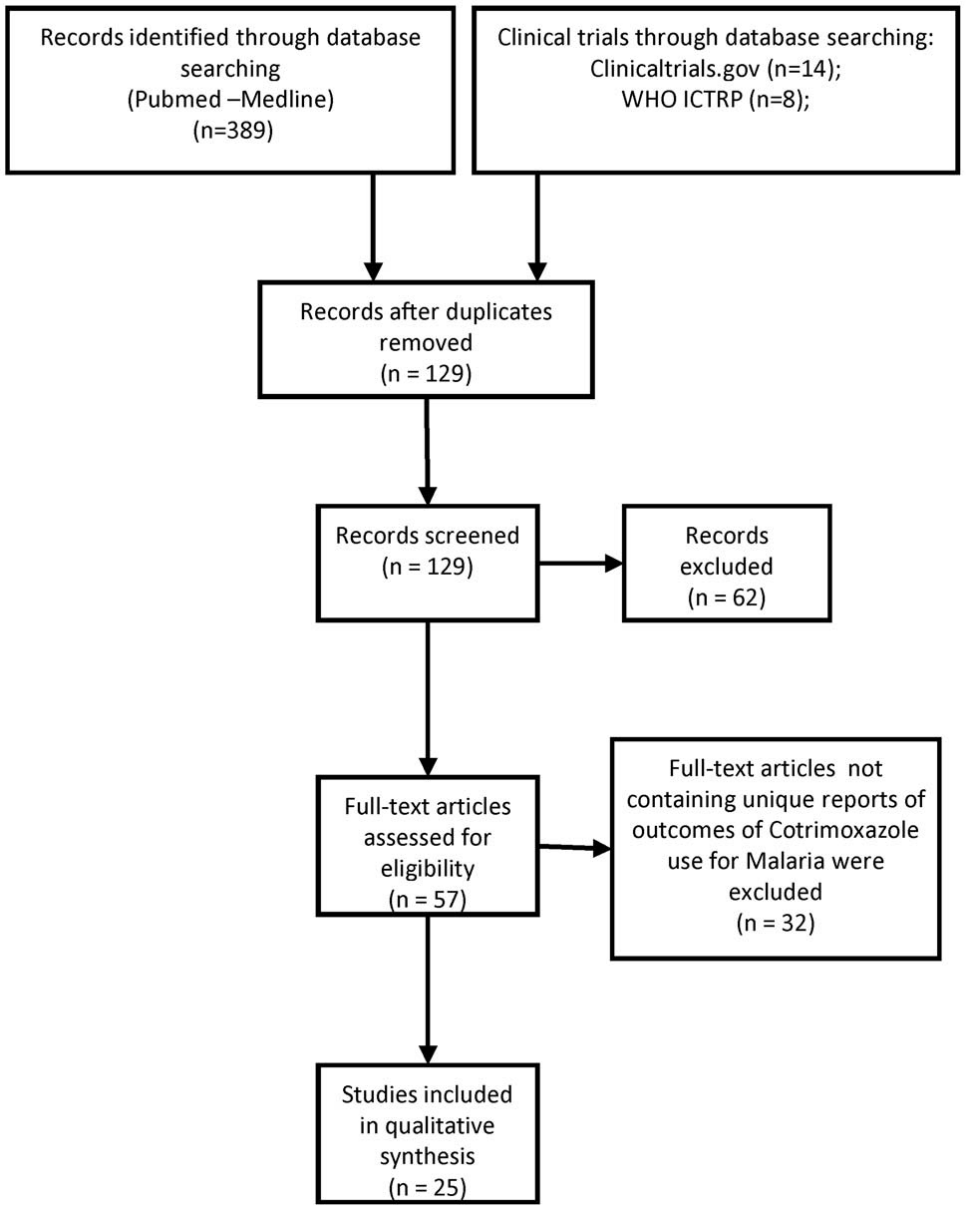
Prisma Flow Chart.

HIV infection, through immune suppression, affects the acquisition and persistence of immune response to malaria. [12] In SSA, HIV infection may cause an average increase of 1·3% in malaria prevalence and of 4·9% in malaria-related mortality [13]. Each year an extra three million clinical malaria cases and 65,000 malaria-related deaths can be attributed to HIV infection. [13] Among HIV infected pregnant women malaria infection [4], [14]–[17] and clinical malaria [18], [19] are more frequent with the latter giving rise to higher parasite densities than those in HIV-uninfected pregnant women. The proportion of placental malaria cases attributable to HIV co-infection increases with the number of pregnancies: 21·3% during the first pregnancy, 41·2% in second pregnancy and 58·2% in third or more pregnancies [20] Further, immunological data indicate that HIV impairs parity-related specific immunity. [5] Cotrimoxazole (CTX) prophylaxis is currently recommended by WHO to prevent opportunistic infections in persons living with HIV/AIDS. [21] In HIV infected children, who are even more vulnerable, daily CTX prophylaxis is also recommended to prevent HIV-related opportunistic infections [22]–[25].

CTX is a drug combination consisting of trimethoprim and sulfamethoxazole. Trimethoprim (2,4-diamino-5-(3,4,5 trimethoxybenzyl pyrimidine)) belongs to a group of compounds with antibacterial and antimalarial activity. It is an inhibitor of dihydrofolate reductase and has been shown to act as a sulfonamide potentiator. [26] The inhibitory action of the combination on bacterial metabolism and in treating bacterial diseases has been well documented. [26], [27] Burroughs Wellcome and Company introduced trimethoprim and sulfamethoxazole (5-methyl-3-sulfanilamidoisoxazole) in the ratio 1∶5. In dealing with bacterial infections, the dosage producing satisfactory treatment contained 320 mg of trimethoprim plus 1·6 g of sulfamethoxazole daily in divided doses for about seven days in adults. [28] A 1∶5 combination of trimethoprim (8 mg/kg bodyweight) and sulfamethoxazole (40 mg/kg bodyweight) was found to effectively treat malaria infections in semi-immune Nigerian children aged 5–12 years. [29] It was also effective in treating chloroquine-resistant *P falciparum* infections. [29] CTX is not gametocytocidal and its sporontocidal activity is unknown. Therefore, Wilkinson and colleagues suggested in 1973, to supplement CTX treatment with an effective gametocytocidal or sporontocidal drug in order to interrupt transmission. [30] More recently, several studies have confirmed higher gametocyte carriage after SP compared to other antimalarial drugs, with peak gametocyte prevalence at around seven days post-treatment. [31]–[35] However, gametocytes present in the peripheral blood after SP treatment seem to have low infectivity for *Anopheles gambiae* sensu stricto (ss) mosquitoes [36]. A similar increase of gametocyte carriage after CTX treatment has been observed. [37] CTX has similar mechanisms of action and resistance patterns to SP and therefore, concerns were raised on the potential impact of CTX resistance on SP efficacy, preventing the implementation of CTX prophylaxis in SSA [38], [39].

CTX is well known as an antibacterial drug but less as an antimalarial. Nevertheless, considering the reports on the impact of CTX on malaria, both in HIV-infected and uninfected individuals, we reviewed the available evidence on safety and efficacy of CTX as an antimalarial for both preventive and curative use.

## Methods

### Study Selection

This systematic review follows PRISMA guidelines. We included all electronically available, peer reviewed articles. We included studies in English as well as abstracts for which a full study was not available such as conference deliberations as long as sufficient data for inclusion were provided. All eligible studies irrespective of sample size were included (see attached flow chart). A protocol for systematic reviews was not used to for this particular review.

### Study Participants

The studies included comprise sample populations who were administered CTX for treatment or prophylaxis or any other drug administered to compare efficacy and safety with that of CTX. The review also includes data from in-vitro studies that involve CTX. The patients involved include children, adults and pregnant women, HIV/AIDs infected and uninfected individuals.

### Search Methods

A literature search was performed to identify publications reporting on safety and efficacy of Cotrimoxazole for malaria treatment or prophylaxis. The search terms included: “Cotrimoxazole prophylaxis and malaria”, “Cotrimoxazole malaria treatment”, “Cotrimoxazole malaria and HIV”, “Cotrimoxazole malaria drug resistance”, “Cotrimoxazole, malaria in pregnancy”, “trimethoprim-sulfamethoxazole and malaria prophylaxis”, “trimethoprim-sulfamethoxazole and sulfadoxine–pyrimethamine”. The search covered the period from 1962 to June 2012. The PubMed was last accessed on June 8^th^, 2012. The same terms were used to search other databases such as the ClinicalTrials.gov and the WHO International Clinical trials Registry Platform (ICTRP). The articles identified were downloaded and reviewed. Studies included were prospective and most of them were done in West Africa, East Africa and a few in Southern Africa. Articles selected were stratified according to the target group (children, non pregnant and pregnant adults) and HIV infected population.

## Results

### Cotrimoxazole for Treatment of Malaria

Several clinical trials reported that CTX was efficacious against *P. falciparum* malaria, both in children and adults and was generally safe as no adverse effects were reported from the studies reviewed (Table 1). In the 70 s and 80 s, CTX was reported to be as effective as chloroquine for treatment of malaria. Parasite clearance rates were similar but fever clearance rates were higher in the chloroquine group due to its antipyretic properties. No recrudescence was observed up to 60 days post-treatment. [30], [40] However, CTX had no gametocytocidal effect. [29] In Tanzania, in the mid 90s, CTX cleared 97% of infections by day seven, while chloroquine only cleared 19%. [41] In 1991 and 1998, both in The Gambia and Uganda, CTX and SP were both effective in reducing fever, clearing parasitaemia and improving anaemia in children less than five years of age with uncomplicated malaria [42], although in Uganda efficacy varied by geographical areas. [43] In 1999, in a hyperendemic area in Southwest Nigeria, both SP and CTX had similar efficacy; and the gametocyte prevalence and parasite density were high for both SP and CTX, though for the latter it was lower than for SP. [44] Later, from 2001 to 2005, in Kenya, Malawi and Nigeria several studies demonstrated that CTX was still efficacious (up to and above 90%) as antimalarial treatment in areas of high endemicity. [37], [44]–[46]. These results reveal that CTX compares with standard treatment of SP for example in the two Kenyan endemic areas of Oyugi in the West and Tiwi in the coast in that their clinical and parasitological failure rates were similar. The combined incidence of parasitological failure over 14 days for the combined sites for CTX was 11% and SP was 16% (RR:0.72, p = 0.29). The 14 day clinical failure rate for the combined sites for CTX was 3.3% and for SP 5.5% (RR:1.69, p = 0.13) [45]. In Malawi in 2001, in the outskirts of Blantyre, an area of high malaria transmission, children were treated for malaria and pneumonia (using Integrated Management of Childhood Illness - IMCI) dual classification). Their clinical, parasitological responses as well as gametocyte prevalence were obtained. The results revealed a total of 78.2% of children receiving CTX and 80.0% receiving SP plus Erythromycine reached adequate clinical and parasitological responses (ACPRs) (p = 0.19) [37]. In a hyper-endemic area of south-western Nigeria, there was 11% treatment failure after 14 days of follow up of uncomplicated *P. falciparum* malaria for CTX which was used for treatment of malaria as was chloroquine, mefloquine, and SP. Independent predictors of failure were age <3 years (adjOR = 0.10; p = 0.007) and body temperature ≥38°C 2 days after the commencement of treatment (adjOR  = 4.9; p = 0.03 [46].

**Table 1:** Summary of prospective studies assessing Cotrimoxazole used for malaria treatment.

Country (author)	Year*	Study population	Type of study	Sample size	Outcome: Efficacy/Safety	Comments
Nigeria (Fasan O et al)[^29^]	1970^p^	5–12 years, school children with asymptomatic parasitaemia	RCT, single dose administered to all children as:(8 mg T & 40 mg SX);(4 mg T &20 mg SX); 15 mgCQ/kg body wtagainst a placebo.FU up to14 days	200	CTX in single dose is efficacious (cleared 100% parasitaemia in less than 72 hrs) against asymptomatic malaria infection and is safe (no adverse reactions reported)	
Thailand (Wilkinson et al)[^30^]	1972^p^	Adults, UM with pre-treatment gametocytaemia	Non-randomized trial, CTX treatment administered 12 hourly for 7·5 days. (FU duration not mentioned)	12	CTX cleared 100% asexual *P. falciparum* parasites (no adverse effects reported)	No gametocytocidal effect
RSA (Hansford et al)[^40^]	1978	all ages exceptpregnantwomen, UM	RCT, CTX *versus*CQ. FU on days 7,12, 42 and 60	63 (19 on stdCTX, 23 on highdose CTX, and21 on CQ)	CTX cleared 100% parasitaemia in less than3 days; CTX high dose (4 tablets twice daily)for 2 days cleared 100% parasitaemia in2·5 days. Efficacy comparable to chloroquine,pyrexia responded slower for CTX. Butno recrudescence in 60 days(noadverse effects reported)	CTX at either dosage appeared to have no effect on gametocytes (by day 21 no gametocytes were detected).
Gambia (Daramola et al)[^42^]	1991^p^	2 groups were studied; Group (1). 7 monthsto 23 months, UM andARI; Group (2). 1 to5·7 years,asymptomaticparasitaemia.	Non-randomized trial, CTX std treatment. FU on days 3, 6 and 21.	Group (1) 10 & Group (2) 65	0% parasitaemia on days 3, 6 and 21except 1 patient who was positive on day 3but reduced parasitaemia and negative onDay 21. 3·3% asymptomatic subjects werepositive but markedly reduced parasites after6 days. (no adverse effectsreported)	Occurrence of low level of R1 resistance to CTX in a rural area was a concern.
Tanzania (Mutabingwa et al)[^41^]	1996^ p^	<5 years, UM, *P.falciparum* monoinfection	RCT, CTX *versus*CQ (25 mgbase/kg over 3days); FU days14, 21 and 28.	61 CTX *vs* 63 CQ	CTX cleared 97% within 7 days compared to CQ which cleared 19%. (no adverse effects reported)	MPCT was 4·3 days for CTX and 5·3 d for CQ. (MPCT was 2·6 d for CTX in 1975 in same area and 2·7 d in 1991 in Malawi). Study conducted in multi-drug resistant *P. falciparum* malaria area.
Uganda (Kilian AHD et al)[^43^]	1996	<5 years, UM, *P. falciparum* monoinfection	Non-randomized trial CTX, with follow-up on days 3, 7 and 14	3 geographical areas: A1∶66; A2∶43 A3∶50	Effectiveness of 40/8 mg/kg CTX differed significantly according to geographical areaby parasitological failure rates (43·9%,34·9% and 10·0% for areas A1., A2. AndA3. respectively). (no adverseeffects reported)	Therapeutic effectiveness is relative to micro-epidemiology resistance
Kenya (Omar et al)[^45^]	1998	<5 years, UM, *P. falciparum* monoinfection	RCT: CTX *vs* SP(one dose). FU ondays 0, 1, 2, 3,and 4.	A1K. CTX 66 andSP 76; A2K CTX57 and SP 69	CTX and SP were both >90% efficaciouswith similar fever clearance time (FCT),Parasite clearance time (PCT) andhaematological findings.(no adverseeffects reported)	Holo-endemic malaria areas in Kenya, CTX use could help to prevent the development of anti-folate resistance strains.
Nigeria (Sowunmi A, et al)[^46^]	1998 & 2003	<13 years UM, *P.falciparum* monoinfection, >1000 asexual forms/µl, no concomitant illness	RCT; CTX *vs* SP or CQ; FU on days1–7 and 14	Only 101 exposed to CTX reported	CTX had 89% efficacy by day 14. Of the 11% failures, predictors of failure were age<3 years and body temperature ≥38°C 2days after treatment commencement.(no adverse effects reported)	Assessed in hyper-endemic area of south-western Nigeria
Nigeria (Sowunmi et al)[^44^]	1999	6 months-12 years,UM, *P. falciparum*malaria, >2,000asexual forms/µl	RCT; CTX *vs* SP	CTX = 53; SP = 49	CTX was 89% and SP 88% efficaciousafter 14 days	Hyperendemic malaria
Malawi (Hamel et al)[^37^]	2001	6 months to 5 years,UM and pneumoniawith *P. falciparum*mono-infection,>2,000 asexualforms/µl	RCT with CTX *vs* SP+E for 5 days;FU 1–4 days anddays 7 and 14	CTX = 104; SP+E = 101	ACPR: 87·2% in CTX; 80% in SP+E; ACR CTX 96·1% and SP+E 88% (p = 0·03); (no adverse effects reported)	The Blantyre District is an area of high *P. falciparum* malaria

* = Year of study

P = Year of publication).

ACPR = Adequate Clinical and Parasitological Response, ACR = Adequate Clinical Response, ARI = Acute Respiratory Infection, A1. =  Bundibugyo area, Uganda. A2. =  Kabarole east area, Uganda. A3.  = Kabarole west area, Uganda. A1K = Tiwi, Kenya, A2K = Oyugis, Kenya. CTX = Cotrimoxazole, CTX standard treatment = (2 tablets twice daily for 5 days), CQ = Chloroquine, E =  Erythromycin, FU = Follow up, Kg =  kilograms, mg = milligrams, MPCT = Mean Parasite Clearance Time, RCT = Randomized clinical trial, SP = sulfadoxine-pyrimethamine, std =  standard, SX = sulfamethoxazole, T = Trimethoprim, UM =  uncomplicated malaria,/µl = per microlitre, vs = versus.

### Cotrimoxazole Malaria Prophylaxis in Non Pregnant HIV Positive Population

Cotrimoxazole prophylaxis is a well established strategy to prevent opportunistic infections in HIV-infected individuals [47]–[49]. Starting Highly Active Anti-Retroviral Therapy (HAART) should not be a reason for not starting or interrupting CTX prophylaxis as this was demonstrated to be beneficial when maintained for more than a year after HAART commencement. [50]. The rationale is that patients with CD4 cell counts >200 cells/µL are still at higher risk of opportunistic infections [51]–[55]. At present, the threshold of CD4 cell counts above which CTX is not advantageous has not yet been identified. [55] However, in the DART trial, carried out in a region where the efficacy of CTX prophylaxis was most questioned, co-administering CTX with HAART halved mortality within the 72-week follow up. The effect in Uganda was sustained beyond 72 weeks, consistent with the reports that CTX is an effective agent for malaria prophylaxis in semi-immune adults [55].

CTX prophylaxis administered to non-pregnant, HIV-infected patients living in an area of moderate, stable malaria transmission reduced the risk of malaria infection (Table 2). In Uganda, 128 HIV-infected adults on HAART and CTX with sustained HIV viral load of ≤400 copies/µL for a period of 4 years had a low risk of malaria infection. [56] In another Ugandan prospective cohort study carried out on HIV infected adults living in a high malaria transmission area, CTX was associated with a 76% lower malaria incidence rate. CTX combined with HAART or with both HAART and insecticide-treated bednets (ITNs), reduced malaria incidence by 92% and 95%, respectively. [47] Also in Uganda, in an area with a high level of anti-folate resistance, ITN use and CTX prophylaxis in HIV infected children reduced malaria incidence by 97%. [25] In another study, during a 6-month follow-up period, patients on CTX prophylaxis and HAART developed no clinical malaria as this finding was attributed to low parasite densities, as parasite densities correlate positively to occurrence of symptoms. [57] Other than long-term HAART, which restores immunity, long term use of CTX prophylaxis is perceived to contribute to the host’s response induced by HAART to achieve the asymptomatic *P. falciparum* parasitaemia. [57] Even when rates of antimicrobial resistance to CTX are high among diarrhoeal pathogens and other bacteria, CTX use is still associated with reduction in mortality and reductions in malaria, diarrhoea, clinic visits and hospital admissions. [58] Apart from reducing HIV morbidity and mortality, an additional advantage of CTX and HAART is malaria prevention and the provision of ITNs reduces the incidence of malaria even further [25].

**Table 2:** Cotrimoxazole for malaria prophylaxis in non pregnant HIV positive population.

Country (author)	Year*	Study population	Type of study	Sample size	Outcome: Efficacy/Safety	Comments
Uganda (Mermin et al) [^68^]	2001 & 2002	HIV-1 infected clients and their HIV negative family members	Prospective cohort study	879 HIV infected participants and 2771 HIV negative family members. HIV infected participants received daily CTX andhousehold family werefollowed up to 17months (+ initial 5months of visits)	CTX prophylaxis taken by HIV persons was associated with decreased morbidity and mortality among HIV negative family members <10 years. Mortality reduced by 63% and malaria was less common (IRR = 0·62 p<0·0001)	Concerns regarding the spread of bacterial resistance should not impede implementation of CTX programs
Uganda (Mermin et al)[^62^]	2001 to 2003	≥5 years and <5 years, on CTX prophylaxis, FU for 1·5 years	Prospective observational cohort study	509 HIV-1 infected and 1522 HIV-negative household members	CTX reduced mortality by 46% and lower rates of malaria (incidence 0·28[0·19–0·40], p<0.0001), diarrhoea(0·65[0·53–0·81] p<0·0001)and hospital admission(0·69[0·48–0·98]. Rareadverse reactions (<2%per person-year)	CD4 annual decline was less and annual increase in viral load was lower. Study conducted in an area with high rates of antimicrobial resistance to CTX
Uganda (Mermin et al)[^47^]	2001; 2001 to2003; 2003 to2004; 2004to 2005	≥18 years	Prospective cohort studydone in 4phases	2001∶466 (of whom 399 on CTX prophylaxis); 2003∶138 survivors from first cohort plus 897new participants onART & CTX, 2004: the participants weregiven ITNs	Baseline: 50 episodes/100 persons per year; CTX prophylaxis only: 76% lower malaria, 9 episodes/100 persons per year; ART+CTX: 92% lower malaria rate (IRR 0·24 [0·15–0·38], p<0.0001; ART, CTX and ITNs: 95% lower malaria rate(IRR 0·05 [0·03–0·08],p<0.0001	Study areas are high –intensity transmission areas.
Uganda & Zimbabwe (Walkeret al)[^55^]	2003 to 2004	Adults ≥18 years, symptomatic AIDS stage2–4, CD4 counts <200/µL,no previous ART apartfrom PMTCT commencedon triple-drug ART.	Observational study from DART trial. CTX prophylaxis was not routinelyused butvariablyprescribed byclinicians. FU wasfrom 4·5 to5·3 years.	3179 participants	CTX prophylaxis reduced mortality (odds ratio: 0·65; p = 0·001) up to 12 weeks; sustained from 12–72 weeksbut not evident subsequently.CTX prophylaxis reducedfrequency of malaria (odds ratio0·74; p−0·0005), maintainedwith time.	CTX prophylaxis for at least 72 weeks for all adults starting combination ART is recommended
Uganda (Nakanjako zt al)[^56^]	2004 to 2005	33–44 years on HAART & on CTX prophylaxis, sustained HIV viral load,<400 copies/ml for4 years,	Prospective observational cohort	128 patients systematically selected	4% had asymptomatic HRP2 antigenaemia in PLHIV onlong term use of HAART and CTX.(no adverse effects reported)	Observation made in low to moderate stable malaria transmission area
Uganda (Kamya et al)^(24)^	2005 to 2006	1–10 years *vs* 1–11 years healthy children;	Prospective cohort study: CTX+ITNs in HIV infected children *vs* ITNs inHealthy children	300 HIV infectedchildren on CTX +ITNs& 561 healthychildren receivedITNs	CTX +ITNs: 97% reduction in malaria incidence (P<0·001).ITN use: 43% reduction inmalaria incidence (P<0·001)	Study conducted in malaria endemic area with high level of molecular markers of antifolate resistance

* = Year of study.

ART =  Anti-Retroviral Therapy, CTX = Cotrimoxazole, DART = Development of Anti-Retroviral Therapy, HAART = Highly Active Anti-retroviral Therapy, HRP = histidine rich protein, HIV = Human Immunodeficiency virus, IRR = Incidence rate ratio, ITNs = Insecticide Treated Nets, PLHIV = people living with HIV/AIDs, PMTCT = prevention of mother to child HIV transmission.

HIV exposed children experience increased morbidity and mortality in their first years of life compared with HIV uninfected children born to uninfected mothers. [59] Also in HIV-infected children, CTX prophylaxis and ITN use reduced malaria incidence dramatically in a highly endemic and highly resistant to antifolate drugs malaria setting. [25] It is important to note that the use of ITNs alone is associated with a 43% reduction in the incidence of malaria. [25], [60], [61] In Uganda, despite high rates of antimicrobial resistance to CTX among diarrhoeal pathogens and other bacteria, CTX prophylaxis was associated with 46% reduction in mortality and lower rates of malaria, diarrhoea, and hospital admissions. Adverse reactions were rare and affected only <2% per person-year and these were mainly muco-cutaneous in nature which resolved with therapy withdrawal. Restarting CTX prophylaxis in 89% of affected individuals, none had any further adverse reactions. [62] The rates of morbidity and mortality reduction in Uganda are similar to those found in other studies in Africa. [63]–[67] In randomized trials done in Uganda and Zimbabwe from 2003 to 2004, CTX prophylaxis significantly reduced mortality and malaria incidence in a sustained manner. [55] Further, another study in Uganda revealed that CTX prophylaxis taken by HIV infected individuals was associated with decreased morbidity and mortality among HIV negative family members [68].

### Cotrimoxazole Malaria Prophylaxis in Non Pregnant HIV Negative Population

There are few studies on CTX prophylaxis in non-HIV infected, and most are reporting secondary analyses of HIV related studies (Table 3). In Mali, CTX had a 99·5% and 97% protective efficacy against symptomatic and asymptomatic malaria infections, respectively. This was observed in an area of low antifolate resistance and selection for SP resistant strains did not seem to occur. CTX was generally safe, as only one patient in the Mali cohort with previous history of Hepatitis A infection and equivocal evidence of past or recent infection with Hepatitis B virus developed acute hepatitis which resolved after withdrawal of CTX. [69] From August 2007 to April 2008, in Tororo, Uganda, an area of extremely high malaria transmission and antifolate resistance, the protective efficacy of daily CTX prophylaxis against malaria in children was 39%. Thus, CTX prophylaxis was moderately protective against malaria in HIV exposed infants when continued beyond the HIV exposure period despite the high prevalence of *Plasmodium* genotypes associated with antifolate resistance. In this particular study, no episodes of skin reactions, allergic drug reactions or other unexpected adverse reactions were reported with CTX administration [70].

**Table 3:** Cotrimoxazole malaria prophylaxis in non pregnant HIV negative population.

Country (Author)	Year*	Study population	Type of study	Sample size	Outcome: Efficacy/Safety	Comments
Mali (Thera)[^69^]	2000	5–15 years in an on-goingcohort study of incidenceof malaria were eligible forinclusion	RCT within Cohort study	160 in CTX group & 80 in the SP group (control). FU periods 11·8 weeks in CTX group and 11·7 weeks inSP group.	From baseline the prophylactic efficacy of CTX against uncomplicated malaria was 99·5% (CI 95: 96%–100%; p<·001) and 97% efficacy against infection.	Seasonal malaria transmission but intense. Study was conducted in peak malaria season
Uganda (Sandison et al)[^70^]	2007 to 2008	6 weeks –9 months,documented HIV uninfectedstatus with mother HIV infected,current breast feeding	Non-blinded RCT: CTX prophylaxis from enrolment until cessation of breast feeding and confirmation of negative HIV status OR uninfected children randomized to stop CTX prophylaxis immediately or continue until 2 years old.	203 breastfeeding HIV exposed infants; 185 HIV negative randomized to stop or continue until 2 years.	CTX when continued beyond the period of HIV exposure = 3.24 cases/person year; When CTX was stopped = 5.57cases/person year.CTX yielded 39% reductionin malaria incidence(IRR 0·61 (95% CI 0·46 to0·81), p = 0·001)	Area of study has high prevalence of *plasmodium* genotypes associated with antifolate resistance.

* = Year of study.

CTX = Cotrimoxazole, FU = Follow up, IRR = Incidence Rate Ratio, PLHIV = people living with HIV/AIDs, RCT = Randomized clinical trial, SP = sulfadoxine pyrimethamine.

### Cotrimoxazole Malaria Prophylaxis during Pregnancy

Numerous studies have demonstrated that HIV infection nearly doubles the risk of placental malaria. [16], [20], [71] Some trials suggest that monthly IPT-SP regimens in HIV infected pregnant women can decrease the risk of placental malaria to levels seen among HIV-uninfected women receiving 2-dose IPT. [72], [73] In HIV infected pregnant women on daily CTX, SP-IPT is not indicated as it may be associated with overlapping toxicities. [74] Fortunately, CTX prophylaxis has shown to decrease the prevalence of placental malaria in HIV infected women as much as IPT-SP in HIV uninfected women (Table 4).

**Table 4:** Cotrimoxazole for malaria prophylaxis during pregnancy in HIV positive population.

Country (Author)	Year*	Study population	Type of study	Sample size	Outcome: Efficacy/Safety	Comments
Malawi (Kapito-Tembo)[^75^]	2005 to 2009	≥15 years, Gestation ≥34 weeks attending routine antenatalservices	Cross sectional study	1121 had data on CTX and/or SP-IPT intake	CTX+SP-IPT: microscopic malaria 0·6%, PCR: 3·6% CTX only: microscopic malaria 2·7%, PCR 5·5% SP only: microscopic malaria 7·7%, PCR 13·5%	
Uganda (Newman)[^84^]	2008 to 2009(HIV infected)2008 (HIVun-infected)	23 to 33 years old PLHIV womenat delivery, 19 to 29 years oldHIV-uninfected women atdelivery	Cross sectional study comparing placental malaria prevalence between HIV-infected women prescribedCTX andHIV-uninfectedwomen prescribedIPT SP	150 HIV-infected women onCTX; 336HIV-uninfectedwomen on SP-IPT	HIV+ CTX: 19% placental malaria smear, PCR positive 6% HIV- SP-IPT: 26%placental malaria, PCRpositive 9%	High malaria transmission area

* = Year of study (if not available P  = Year of publication).

CTX = Cotrimoxazole, HIV = Human Immunodeficiency virus, PCR = Polymerase Chain Reaction, PLHIV = people living with HIV/AIDs, SP-IPT = sulfadoxine-pyrimethamine-Intermittent Preventive Treatment.

A Malawian study observed a superior efficacy of CTX with or without SP-IPT compared to SP-IPT alone in reducing prevalence of microscopic and PCR-detected malaria infections and anaemia in HIV-infected pregnant women. [75] When taken in the first trimester, CTX intake in pregnancy has been associated with increased risk of folate deficiency, maternal anaemia and poor birth outcomes [76]–[79] and neural birth defects [80]–[82]. However, in the same Malawian study, CTX, with or without SP-IPT, was associated with reduced prevalence of maternal anaemia and higher haemoglobin concentration, consistent with beneficial effects in birth outcomes as previously reported in a Zambian study. [83] This further underscores the fact that for this target group, CTX prophylaxis may be beneficial. Therefore, Newman et al concluded that daily CTX can decrease the risk of placental malaria in HIV infected women [84].

Most studies have demonstrated CTX not associated with hyperbilirubinemia when administered to mothers during pregnancy and breast feeding. No cases of kernicterus were reported in neonates after maternal ingestion of sulfonamides [74], [85], [86]. Further, the database on drugs and lactation revealed that although CTX is detected in breast milk, exposure through breast milk appears to be safe in healthy breastfed infants. [87] Further, a manifestation of small for gestational age was mentioned in a recent study of women exposed to CTX during second and third trimesters compared to those exposed to other urinary antimicrobials and this was classified as uncommon in frequency [86], [88].

There is currently no information on the use of CTX as malaria prophylaxis in HIV-uninfected pregnant women.

### CTX Resistance Versus SP Resistance in *P. falciparum*

The dihydrofolate reductase (dhfr)/dihydropteroate synthetase (dhps) quintuple mutant is known to be associated with SP treatment failure. It is however unclear whether the quintuple mutant affects the protective efficacy of CTX against malaria. [89]–[91] A study in Uganda carried out between 2008 and 2009 found a similar prevalence of placental malaria and resistance markers (*dhfr*-59; *dhps*-437 or *dhps*-540E) in HIV-infected pregnant women taking CTX and HIV–uninfected women taking SP-IPTp [84].

Considering the possible cross resistance between CTX and SP, [92], [93] there have been concerns on the impact of widespread CTX prophylaxis on the selection of SP resistant malaria parasites. [38], [39] Nevertheless, recent studies in Tororo and Kampala, Uganda, found no association between CTX use and increased prevalence of mutations conferring antifolate resistance in HIV infected children and adults taking daily CTX prophylaxis [94], [95] (Table 5).

**Table 5:** *P. falciparum* malaria – CTX resistance or CTX resistance versus SP resistance.

Country (Author)	Year*	Study population	Type of study	Sample size	Outcome: Efficacy/Safety	Comments
Liberia (Petersen 1987)[^98^]	1987	*Plasmodium falciparum*isolates were tested against sulfadoxine, sulfamethoxazole, pyrimethamine and trimethoprim	In vitro susceptibility testing	Two isolates F32 (from Tanzania and sensitive to chloroquine and pyrimethamine) & K1 (from Thailand and resistant to Chloroquine and pyrimethamine)	The difference in IC_50_ betweenF32 and K1 against trimethoprimand CTX was much less than the difference between the IC_50_values against pyrimethamineand SP.	Cross resistance between pyrimethamine and trimethoprim exists but is not complete.
Uganda (Malamba 2010)[^95^]	2001	3 to 34·5 years HIV infected adults and children	Prospective cohort studyadministeringCTX prophylaxis	3,601 blood smears(2,154 taking taking CTX prophylaxis and 1,447not taking CTX	HIV infected taking CTX: dhfrtriple mutant: 74% dhpsmutant: 95% dhfr/dhps quintuplemutant 6: 73% HIV infected not onCTX dhfr triple mutant:70%(P = 0·71) dhps mutant: 88%(p = 0·21) dhfr/dhps quintuplemutant: 64% (p = 0·36).	Extremely high malaria transmission area.
Uganda (Malamba et al)[^96^]	2001 and 2002	≥5 years and <5 years, HIV uninfected household members of PLHIV taking or not taking CTX.	Prospective cohort study	1,319 HIV-uninfected household members of PLHIV taking CTX; 1,248 HIV uninfectedhousehold members ofPLHIV not taking CTX.	Proportion of malaria episodes caused by SP-resistant parasites; HIV un-infected persons = HIV-infected household members not taking CTX. (Overall incidence of malaria [IRR = 0·67, 95%CI = 0·49–0·92])	No evidence that CTX prophylaxis lead to the spread of SP resistant malaria parasites among household members not taking the drug.
Kenya (Hamel et al)[^97^]	2002 to2003	≥15 years, not severely ill,not taking daily antibioticsfor treatment of a chronicillness (excludingtuberculosis)	Prospective study to assess whether the use of dailyCTX resulted in significant changes in antifolate and CTX resistance among common organisms	3 study arms: 132 HIV negative 336 HIV-positive with CD4≥350/µL received daily vitamins 692 HIV-positive with CD4<350 received daily CTX; median FU 24weeks	Daily CTXdid not result in increased *P.falciparum* antifolate resistance;reduced malaria incidence by89–90%Contributed to increased pneumococcus and commensalE. coli resistance (in lowerCD4 subjects P<0.005)	There is need for surveillance with regard to CTX resistance among respiratory and diarrhoeal disease pathogens
Uganda (Gasasira et al)[^94^]	2004 to2005 &2005 to2006	HIV-infected children 1–10 years and healthy children1–11 years;	2 prospective cohort studies:ITN +CTX and ITN	292 HIV-infectedchildren; Duration of FU: 0to 2·4 years 517uninfected children;Duration of FU = 0·2 to2·4 years HIVuninfected	CTX gave 80% protective efficacy and this did not vary over 3 consecutive (9·5 month) periods; Prevalence of dhfr 164L mutation was higher in parasites from HIV infected compared to HIV uninfected children (8% *vs* 1%, p = 0·001)	Study conducted in an area of widespread antifolate resistance.

* = Year of study.

CTX = Cotrimoxazole, DHFR = Dihydrofolate reductase, DHPS = Dihydropteroate synthetase, E. coli =  Escherichia coli, FU = Follow up, HIV = Human Immunodeficiency Virus, IRR = Incidence Rate Ratio, PLHIV = people living with HIV/AIDs, RCT = Randomized clinical trial, SP = sulfadoxine pyrimethamine.

In Uganda, CTX prophylaxis in HIV-infected individuals did not increase the occurrence of SP-resistant malaria episodes among HIV-uninfected individuals living in the same household. Instead, the latter had a lower incidence of malaria and infections with SP resistant parasites. [96] In Kenya, a prospective study observed that daily CTX increased carriage rates of non-susceptible pneumococci and CTX resistant *E.coli* and therefore may accelerate the development of CTX resistance among respiratory and diarrhoeal pathogens, especially in areas with low CTX resistance at baseline. [97] However, regarding malaria, this study demonstrated that CTX prophylaxis reduced the incidence of malaria and antifolate resistant genotypes.

*In-vitro* susceptibility testing against sulfadoxine, sulfamethoxazole, pyrimethamine and trimethoprim on *P. falciparum* isolates from Tanzania and from Thailand revealed incomplete cross-resistance between pyrimethamine and trimethoprim. The cross resistance between CTX and SP did not appear to be absolute at the time of testing [98].

### CTX Resistance of Pathogens Other than Plasmodium

Cotrimoxazole is often used to treat pneumoccal infections. There were concerns related to the use of CTX and SP regarding increased resistance in pathogens other than pneumococci, such as *Haemophilus influenzae* and dysentery-causing bacteria. [99] However, a study done in Uganda reported by Mermin (2005) revealed that daily CTX prophylaxis, taken by persons with HIV, was associated with decreased morbidity and mortality among family members. Antimicrobial resistance among diarrhoeal pathogens infecting family members did not increase. Concerns regarding the spread of bacterial resistance should therefore not impede implementation of CTX program [68].

## Discussion

Artemisinin Combination Therapies (ACTs) are very efficacious and are currently the mainstay for malaria treatment. However, for prophylaxis the options are limited. Despite the high and increasing drug resistance, only SP is at present eligible to be used for IPTp and IPTi. [100] It is unclear what could be a valid alternative to SP as newer drugs are still in the pipeline of development to be used for IPTp such as Mefloquine, Dihydroartemisinin-piperaquine, chloroquine-azithromycin and SP-azythromycin. This review indicates that CTX may be a possible alternative as there is a long history of CTX remaining efficacious and safe for malaria treatment and prophylaxis for different target groups.

The wealth of information available on the use of CTX in HIV infected people confirms that CTX is effective against malaria infection and in preventing clinical malaria. Further, despite its long term use, CTX is not associated with a higher prevalence of mutations related to antifolate resistance and works synergistically with ITNs in preventing malaria. Therefore, CTX can be successfully used to prevent HIV/AIDS-related opportunistic infections and malaria morbidity, an important feature in sub-Saharan Africa where these two diseases co-exist and health services operate under constrained budgets. Besides being relatively safe CTX is also inexpensive, almost universally available and has a wide clinical spectrum of activity spanning from bacteria, fungi and protozoan infections [101].

The safety concerns for CTX is related to its impact in malnourished individuals in whom it may precipitate folate deficiency and pancytopaenia. [102] Common gastrointestinal disturbances include nausea and vomiting and less commonly diarrhoea. Cholestatic jaundice had also been documented. Sulfamethoxazole is known to cause headache, depression and hallucinations. [102] Hypersensitivity reactions like neutropenia, Stevens-Johnson syndrome (SJS) and Sweet’s syndrome occur more often in HIV/AIDs patients. [102] The reactions in this group have been related to their poor ability to handle nitroso-derivatives of sulfamethoxazole. [103], [104] Generally, the wide use over time has proven CTX to be safe.

The fact that in pregnant women CTX prophylaxis showed a similar prevalence of placental malaria in HIV infected women as IPT-SP in HIV uninfected women suggests that daily CTX can similarly decrease the risk of placental malaria. In HIV-infected pregnant women, CTX use was associated with decreased malaria infection, maternal anaemia and increased haemoglobin concentration, a finding consistent with the beneficial effects on birth outcomes. Therefore, CTX may have similar beneficial effects in other groups though there is currently no data available on its use as a malaria preventive measure in HIV uninfected pregnant women or children. The studies reporting on CTX prophylaxis in HIV infected pregnant women were cross-sectional studies and there could be residual confounding from unmeasured factors. Further, participants were only enrolled in the third trimester of pregnancy [75] and at delivery [84] and therefore the overall impact of CTX on malaria infection and anaemia may be underestimated. Despite these limitations, these studies provide important data on the impact of CTX prophylaxis on the epidemiology and clinical implications of placental malaria among HIV-infected women.

A limitation of this review is that most information available comes from East and Western Africa. Almost all cited publications are published in English and relevant literature in other languages may have been overlooked. Nevertheless, this review identified the need of determining the benefits of using CTX in HIV-uninfected risk groups, such as children and pregnant women. The constraints related to the use of CTX may relate to the fact that it should be taken daily and issues related to its acceptability, safety, adherence and potential selection of resistant strains including antibiotic resistance.

### Conclusions

CTX has been extensively used for half a century as an antibiotic worldwide and in malaria endemic areas. CTX antimalarial effect, although scientifically proven, has been ignored. Its long term use in HIV infected children and adults, has proved that CTX is still effective for malaria prevention and treatment. This has been confirmed in a few studies in non-HIV infected population, mostly secondary analyses of HIV studies. More information is required for pregnant women, irrespective of HIV infection for whom no information is available. There is need for more randomized controlled trials to evaluate further the efficacy of CTX as antimalarial, since most of the available data are derived from descriptive studies.

Research on CTX safety, adherence and acceptability is still necessary if its role for malaria treatment and prophylaxis in groups other than HIV-infected individuals is to be established.
